# Click-code-seq protocol for genome-wide single-nucleotide-resolution mapping of major types of DNA modifications

**DOI:** 10.1016/j.xpro.2026.104776

**Published:** 2026-08-10

**Authors:** Lucie Lefevre, Emilie A. Aghajani, Navnit K. Singh, Livia Maria Anna Pietrow, Nikolai J.L. Püllen, Vakil Takhaveev, Shana J. Sturla

**Affiliations:** 1 Department of Health Sciences and Technology, ETH Zurich, 8093 Zurich, Switzerland

**Keywords:** Bioinformatics, Sequence analysis, Genomics, Sequencing, Molecular Biology

## Abstract

DNA modifications are central to carcinogenesis, chemotherapy drug action, neurodegeneration, and aging. Here, we present a protocol for click-code-seq, a next-generation sequencing technique for genome-wide single-nucleotide-resolution mapping of major types of DNA modifications: oxidized guanines and abasic sites. We describe steps for genomic DNA extraction and library preparation, including enzymatic modification conversion and click-chemistry-based tagging. We then detail procedures for data analysis using the associated computational pipeline Click-pipe.

For complete details on the use and execution of this protocol, please refer to Takhaveev et al.^1^

## Highlights

• Reduction of background signal from non-targeted DNA modifications
• Enzymatic excision of oxidized guanines and abasic sites into single-nucleotide gaps
• Gap filling and tagging with MoDIS barcodes by click chemistry
• Computational pipeline Click-pipe for DNA-modification calling

## Abstract (editor highlights)

Publisher’s note: Undertaking any experimental protocol requires adherence to local institutional guidelines for laboratory safety and ethics.

## Graphical abstract

**Figure:**
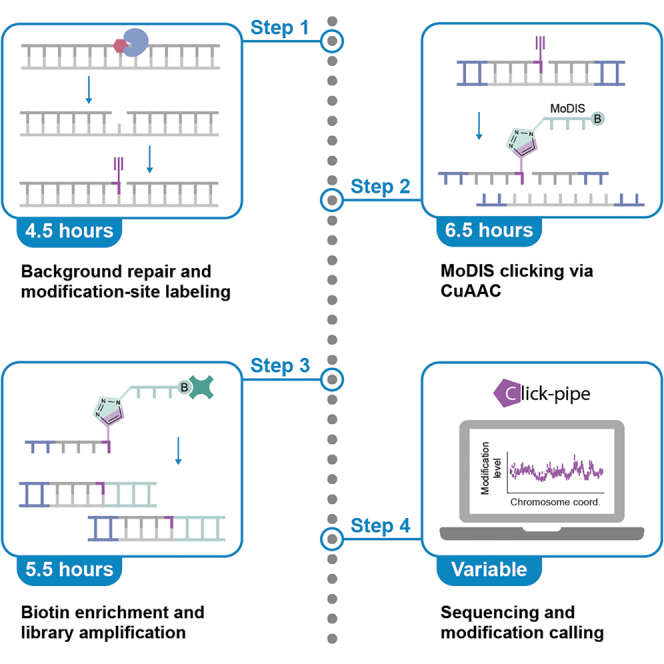

## Before you begin

Click-code-seq enables single-nucleotide-resolution mapping of 8-oxoguanine (8-oxoG) and abasic (AP) sites in large genomes such as the human genome. Most previous antibody-based or chemical enrichment methods to map DNA modifications are limited to low-resolution maps in the 0.1 to 1,000 kb range.^2^^,^^3^^,^^4^ While several approaches for base-resolution mapping of oxidized guanines and AP sites have now been reported, including CLAPS-seq,^5^ CD-seq,^6^ and nanopore-based detection^7^ for oxidized guanines, and snAP-seq^8^ and SSiNGLe-AP^9^ for AP sites, click-code-seq offers several advantages. For example, it encodes modification positions prior to fragmentation, reducing handling-induced artifacts. It uniquely incorporates a background reduction step that ensures high specificity. Modification positions are directly encoded through gap filling and molecular tagging rather than inferred from polymerase stalling or DNA-break patterns, enabling stringent filtering of amplification artifacts and molecule-level quantification through PCR duplicate removal. Finally, click-code-seq covers the mappable genome without sequence-context restrictions, unlike the nanopore approach, which is currently limited to ∼35% of guanines in the human genome.^7^

**Figure 1:**
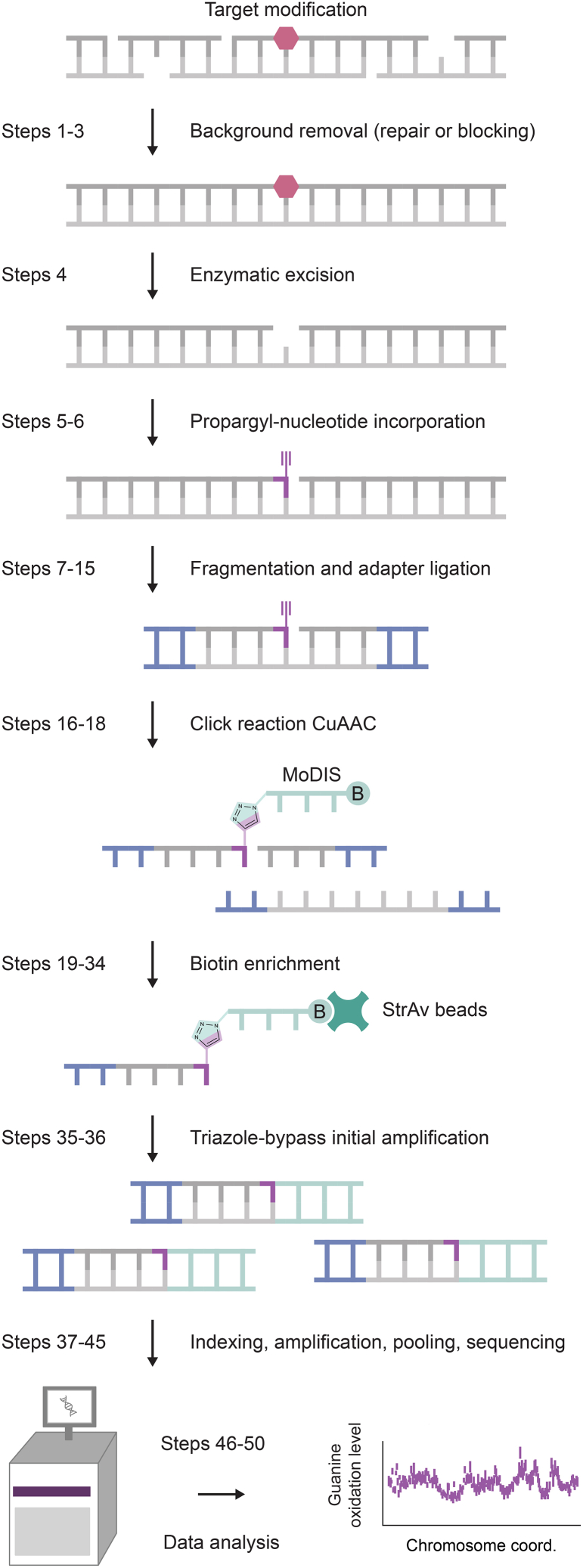
Overview of the click-code-seq workflow Genomic DNA is extracted and subjected to enzymatic removal of pre-existing background modifications prior to modification-specific labeling. When mapping oxidative DNA modifications, background modifications include abasic sites, single-strand breaks and gaps. When mapping abasic sites, background modifications include single-strand breaks and gaps. Target DNA modifications are enzymatically excised, generating defined 3′-OH termini, which are filled by incorporating propargyl-modified nucleotides to encode modification positions. Labeled DNA is fragmented and sequencing adapters are ligated. A copper-catalyzed click reaction covalently attaches a biotinylated Modified DNA Identifier Sequence (MoDIS) oligonucleotide to the propargyl-labeled sites, followed by streptavidin-based enrichment to remove unlabeled fragments. An initial amplification step using a triazole-bypass polymerase enables copying across the click-generated linkage. Libraries are then indexed, amplified, pooled, sequenced and processed through a dedicated data-analysis pipeline to generate genome-wide single-nucleotide-resolution maps of DNA modifications. StrAv: Streptavidin.

The protocol below describes specific steps for mapping oxidized guanines using formamidopyrimidine DNA glycosylase (Fpg) and endonuclease IV (EndoIV), and for mapping abasic sites using EndoIV alone. In principle, the protocol can be adapted to any DNA modification for which there is an enzyme capable of excising it, resulting in a single-nucleotide gap with a 3′-OH end. For example, alkyl adenine DNA glycosylase (hAAG) does this with alkylated bases. The protocol is compatible with fresh or frozen cultured cells and tissues and, in principle, any biological source from which DNA can be isolated. Click-code-seq can be used to detect endogenous modifications as well as damage induced by chemical exposure or other perturbations *in vitro* and *in vivo*. The resulting genome-wide, single-nucleotide-resolution maps provide insight into the genomic distribution of DNA modifications, their sequence-context preferences, their relationship to mutational signatures, and the influence of transcription and replication on modification patterns and repair. An overview of the complete click-code-seq workflow is provided in Figure 1, and a detailed illustration of the library construction steps and oligonucleotide sequences is provided in Figure S1.

### Innovation

Click-code-seq uses click chemistry to covalently tag DNA modification sites that have been permanently encoded through enzymatic excision and propargyl-nucleotide incorporation.^10^ Each modification site is enzymatically converted into a single-nucleotide gap filled with 3′-*O*-propargyl-modified nucleotides by Therminator IX polymerase, a variant engineered for unnatural substrates. Copper-catalyzed azide–alkyne cycloaddition (CuAAC) forms a biocompatible triazole linkage connecting a code sequence to the propargyl-labeled site, allowing Vent (exo-) DNA polymerase to read through the labeled site during library amplification. Additionally, since target modification positions are encoded prior to DNA fragmentation, false-positive signals from sonication-induced DNA damage are avoided. Finally, since pre-existing 3′-OH termini at strand breaks, as well as abasic sites susceptible to endonuclease cleavage during oxidized guanine mapping, can incorporate propargyl-modified nucleotides and produce false-positive signals, the protocol includes an enzymatic repair or blocking step prior to excision. This establishes a defined baseline in which only target modifications are converted into sequencing signals.

Another key innovation of this protocol is the design of the code sequence MoDIS (Modified DNA Identifier Sequences), which addresses the increased complexity of large mammalian genomes compared to smaller genomes and ensures quantitative accuracy of individual modification events. MoDIS incorporates two functional elements: a validation code (VC), which is a fixed nucleotide sequence that must be present in the read to pass filtering, thereby removing non-specific PCR amplification artifacts; and a randomized index code (RIC), which functions as a unique molecular identifier (UMI) to distinguish identical modifications occurring in different cells and chromosomes and enable PCR duplicate removal during computational deduplication.^1^

### Preparation of adapter and MoDIS mixes

**Timing: 1 h**

1. Prepare the 15 μM annealed P7_prox_X duplex:
  a. Add 15 μL of P7_prox_Xa (100 μM), 15 μL of P7_prox_Xb (100 μM), 10 μL of 10x annealing buffer, and 60 μL of elution buffer (EB; 10 mM Tris-HCl, pH 8.5).
  b. Heat the mixture to 90 °C for 3 min, then cool to 25 °C over 45 min in a thermocycler.

***Note:*** Prepare four separate duplexes, one for each of X = 1, 2, 3, and 4 (Figure S1). Store at −20 °C for up to 2 years.

2. Prepare the 15 μM P7 proximal adapter mix by combining equal volumes of annealed P7_prox_1, P7_prox_2, P7_prox_3, and P7_prox_4 duplexes (15 μM each).

***Note:*** Store at −20 °C.

3. Prepare the 250 μM P5 proximal MoDIS mix by combining equal volumes of MoDIS_1 (250 μM) and MoDIS_2 (250 μM).

***Note:*** Store at −20 °C for up to 2 years.

### Extraction of genomic DNA

**Timing: 1 h**

4. Extract DNA using NEB Monarch kit following manufacturer’s instructions available here.
5. Elute the DNA from the column using EB (10 mM Tris-HCl, pH 8.5).

**CRITICAL:** Do not use the elution buffer provided in the NEB Monarch kit because it contains EDTA, which will inhibit EndoIV, *Bst* DNA polymerase, and *Taq* DNA ligase in the background repair step, as well as Fpg and Therminator IX in subsequent steps.

***Note:*** Other kits for genomic DNA extraction can be used. The NEB Monarch kit is recommended for short lysis and incubation times to minimize artifactual DNA oxidation during sample preparation.

**CRITICAL:** Proceed directly with the step-by-step procedure or store the DNA at −20 °C for up to 2 days or −80 °C for long-term storage. We typically aim to process genomic DNA into libraries within 1–3 months after extraction. However, storage at −80 °C has been reported to preserve DNA integrity for at least one year with no change in DNA methylation and hydroxymethylation levels,^11^ and for at least 10 years based on DNA integrity number (DIN) as endpoint.^12^

## Key resources table

**Table:** 

REAGENT or RESOURCE	SOURCE	IDENTIFIER
**Chemicals, peptides, and recombinant proteins**		

(+)-Sodium L-ascorbate	Sigma-Aldrich	Cat#A4034
3′-*O*-Propargyl-dGTP (prop-dGTP)	Jena Bioscience	Custom synthesis
3′-*O*-Propargyl-dATP (prop-dATP)	Jena Bioscience	Custom synthesis
Disodium hydrogen phosphate dihydrate (Na_2_HPO_4_-2H_2_O)	Sigma-Aldrich	Cat#71643
Sodium dihydrogen phosphate monohydrate (NaH_2_PO_4_-H_2_O)	Sigma-Aldrich	Cat#71507
Copper(II) sulfate pentahydrate (CuSO_4_)	Sigma-Aldrich	Cat#C8027
Tris(3-hydroxypropyltriazolylmethyl)amine (THPTA)	Lumiprobe	Cat#F4050
Dimethyl sulfoxide (DMSO)	Sigma-Aldrich	Cat#W387520
Deoxynucleotides (dNTPs) solution mix	NEB	Cat#N0447S
β-Nicotinamide adenine dinucleotide (NAD^+^)	NEB	Cat#B9007S
Magnesium Sulfate (MgSO_4_) Solution	NEB	Cat#B1003S
Tween 20	Sigma-Aldrich	Cat#P9416
Ethanol	Merck Millipore	Cat#1.100983.1011
UltraPure 1M Tris-HCl, pH 8.0	Invitrogen	Cat#15568025
UltraPure 0.5M EDTA, pH 8.0	Invitrogen	Cat#15575020
Sodium chloride (NaCl)	Sigma-Aldrich	Cat#S3014
Formamidopyrimidine DNA glycosylase (Fpg)	NEB	Cat#M0240S
Endonuclease IV (EndoIV)	NEB	Cat#M0304S
T4 Polynucleotide Kinase (T4 PNK)	NEB	Cat#M0201S
Therminator IX DNA polymerase	NEB	Custom synthesis
*Bst* DNA Polymerase, Full Length	NEB	Cat#M0328S
*Taq* DNA Ligase	NEB	Cat#M0208S
Vent (exo-) DNA polymerase	NEB	Cat#M0257S
NEBuffer 2 (10×)	NEB	Cat#B7002S
ThermoPol Buffer (10×)	NEB	Cat#B9004S
Dynabeads MyOne Streptavidin C1	Life Technologies	Cat#65001
Elution buffer (EB; 10 mM Tris-HCl, pH 8.5)	Qiagen	Cat#19086

**Critical commercial assays**		

Monarch Genomic DNA Purification Kit	NEB	Cat#T3010L
ProNex Size-Selective Purification System	Promega	Cat#NG2001
NEBNext Ultra II DNA Library Prep Kit for Illumina	NEB	Cat#E7645S
NEBNext Multiplex Oligos for Illumina (Index Primers Set 1)	NEB	Cat#E7335S
QuantiFluor ONE dsDNA System	Promega	Cat#E4870
D1000 Reagents	Agilent Genomics	Cat#5067-5583
D1000 ScreenTape	Agilent Genomics	Cat#5067-5582
HS D1000 Reagents	Agilent Genomics	Cat#5067-5585
HS D1000 ScreenTape	Agilent Genomics	Cat#5067-5584

**Deposited Data**		

human reference genome UCSC GRCh38/hg38	UCSC Genome Browser	https://genome-idx.s3.amazonaws.com/bt/GRCh38_noalt_as.zip
mouse reference genome UCSC GRCm39/mm39	UCSC Genome Browser	https://genome-idx.s3.amazonaws.com/bt/GRCm39.zip

**Oligonucleotides**		

P7_prox_1a (representative example): AGACGTGTGCTCTTCCGATCTA GAAGGCCTAG∗T where the asterisk denotes a phosphorothioate linkage; see Figure S1 for complete P7_prox duplex sequences	this paper	N/A
MoDIS_1: N_3_-TNNNNNNNNNNC AACAAAGATCGGAAGAGCGT CGTGTAGG-TEG-Biotin	Takhaveev et al.^1^	N/A
MoDIS_2: N_3_-TCAACAANNNN NNNNNNAGATCGGAAGAG CGTCGTGTAGG-TEG-Biotin	Takhaveev et al.^1^	N/A
P7_prox_primer: AGACGTGTGCTCTTCCGATCT	this paper	N/A
P5_prox_primer: CCTACACGACGCTCTTCCGATC	Takhaveev et al.^1^	N/A
i5/i7 indexing primers: see NEBNext Multiplex Oligos for Illumina (Index Primers Set 1) product manual	NEB	Cat#E7335S

**Software and algorithms**		

click-code-seq preprocessing pipeline (Click-pipe)	this paper	https://gitlab.ethz.ch/eth_toxlab/click_pipe.git
FastQC	Andrews (2010)	https://www.bioinformatics.babraham.ac.uk/projects/fastqc/
MultiQC	Ewels et al.^13^	https://multiqc.info/
Trimmomatic	Bolger et al.^14^	http://www.usadellab.org/cms/?page=trimmomatic
BBMap	Bushnell (2014)	https://sourceforge.net/projects/bbmap/
Cutadapt	Martin^15^	https://cutadapt.readthedocs.io
Bowtie2	Langmead and Salzberg^16^	http://bowtie-bio.sourceforge.net/bowtie2/
Samtools	Li et al.^17^	http://samtools.sourceforge.net/
Bedtools	Quinlan and Hall^18^	https://bedtools.readthedocs.io
UMI-tools	Smith et al.^19^	https://github.com/CGATOxford/UMI-tools

**Other**		

8-strip PCR tubes	Bioconcept	Cat#3131-00
LoBind tubes, 1.5 mL	Eppendorf	Cat#0030 108.116
LoBind tubes, 0.5 mL	Eppendorf	Cat#0030 108.094
Individual PCR tubes, 0.2 mL, flat cap	Thermo Fisher	Cat#AB0620
DynaMag-2 Magnetic Rack	Thermo Fisher	Cat#12321D
Quantus Fluorometer	Promega	Cat#E6150
T100 Thermocycler	Bio-Rad	Cat#1861096
Q800R2 Sonicator	QSonica LLC	Cat#Q800R2
Agilent 4150 TapeStation	Agilent Genomics	Cat#G2992AA
GeneVac SpeedVac	GeneVac	Cat#11574604
Mini centrifuge LLG-uni*CFUGE* 2	LLG-Labware	Cat#6.263 510

***Note:*** All oligonucleotides should be synthesized on a 0.2-μmol scale and purified by reverse-phase HPLC. They should be resuspended in EB (10 mM Tris, pH 8.5). Avoid Tris-EDTA buffer or any other buffer containing EDTA to prevent enzymatic inhibition.

***Alternatives:*** Most chemicals and instruments can be purchased from alternative suppliers as long as the specifications match. However, we have not tested substitutions and recommend using the indicated sources for all enzymes (Fpg, EndoIV, T4 PNK, Therminator IX, *Bst* DNA Polymerase, *Taq* DNA Ligase, Vent (exo-) DNA Polymerase), custom-synthesized nucleotides (prop-dGTP and prop-dATP), and critical commercial assay kits (ProNex Size-Selective Purification System, Monarch Genomic DNA Purification Kit, NEBNext Ultra II DNA Library Prep Kit, and Dynabeads MyOne Streptavidin C1). For DNA quantification, a Qubit fluorometer (Thermo Fisher) is an acceptable alternative to the Quantus fluorometer. For fragment size analysis, an Agilent Bioanalyzer is an acceptable alternative to the Agilent 4150 TapeStation.

## Materials and equipment

### 10× annealing buffer, 10 mL

Dissolve 58.4 mg of NaCl in 8 mL of ultrapure water (e.g., MilliQ, 18.2 MΩ·cm). Add 100 μL of Tris-HCl (1M, pH 8.0). Bring to 10 mL with ultrapure water. Store at 20–25 °C for up to 1 year.

### CuSO_4_ solution, 20 mM, 10 mL

Dissolve 50 mg of copper(II) sulfate pentahydrate (MW = 249.69 g/mol) in 10 mL of ultrapure water to obtain a 20 mM solution. Aliquot and store at −20 °C for up to 1 year.

### THPTA solution, 200 mM, 1 mL

Dissolve 87 mg of THPTA (MW = 434.53 g/mol) in 1 mL of ultrapure water to obtain a 200 mM solution. Aliquot and store at −20 °C for up to 1 year.

**Table:** 2× Bind and Wash buffer

Reagent	Final concentration	Amount
NaCl	2 M	5.84 g
Tris-HCl (1 M)	10 mM	500 μL
EDTA (0.5 M)	1 mM	100 μL
Tween 20	0.1%	50 μL
Ultrapure water	N/A	Up to 50 mL
**Total**	**N/A**	**50 mL**

Store at 20–25 °C for up to 1 year.

**Table:** 1× Bind and Wash buffer

Reagent	Final concentration	Amount
2× Bind and Wash buffer	1×	25 mL
Tween 20	0.1%	25 μL
Ultrapure water	N/A	25 mL
**Total**	**N/A**	**50 mL**

Store at 20–25 °C for up to 1 year.

**Table:** NaH_2_PO_4_ buffer, pH 7, 1 M, 50 mL

Reagent	Final concentration	Amount
Na_2_HPO_4_-2H_2_O	620 mM	5.52 g
NaH_2_PO_4_-H_2_O	380 mM	2.62 g
Ultrapure water	N/A	Up to 50 mL
**Total**	**N/A**	**50 mL**

Adjust pH to 7 using NaOH or HCl, and store at 20–25 °C for up to 1 year.

**CRITICAL:** Do not replace NaH_2_PO_4_ buffer with KH_2_PO_4_ or any other buffer. The protocol was developed and validated exclusively with NaH_2_PO_4_ buffer, and substitution has not been tested.

## Step-by-step method details

***Note:*** The step-by-step method details below describe the protocol for mapping oxidized guanines. For mapping abasic sites, refer to the Alternatives notes in the Background repair and Enzymatic modification excision and propargyl-nucleotide incorporation sections for the relevant modifications to the protocol.

### Background repair

**Timing: 2.5 h**

This step removes background strand breaks and abasic sites (when mapping oxidized guanines) from genomic DNA prior to modification-specific labeling, ensuring that only target modifications are converted into sequencing signals.

***Note:*** The steps below describe the background repair strategy, which is recommended for most applications. Background blocking is an alternative strategy that can be employed instead; for details see Takhaveev et al.^1^

**CRITICAL:** From this point until step 5.d, keep samples on ice during processing to prevent additional 8-oxoG formation. To avoid artifactual damage, use wide-bore tips when transferring DNA and keep pipetting to a minimum. For mixing, flick the tube rather than pipetting.

***Note:*** Unless specified otherwise, perform any of the following steps in either 1.5 mL DNA LoBind microcentrifuge tubes or 0.2 mL PCR tubes/strips. Use a magnetic stand appropriate for the chosen tube type during bead-based purification steps.

1. Prepare the DNA samples by pipetting the volume corresponding to 2.5 μg of DNA and filling with ultrapure water to 14.5 μL.
2. Perform the background repair reaction:
  a. Prepare a master mix containing the reagents below and mix well by pipetting.
    ***Note:*** Multiply the volumes by the number of reactions that are to be performed plus one in excess to ensure that the master mix suffices for all samples.

**Table:** 

Reagent	Volume for a single reaction (μL)	Final concentration^a^
ThermoPol buffer (10×)	3	1×
NAD+ (5 mM)	3	500 μM
dNTPs (10 mM)	3	1 mM
EndoIV (10 U/μL)	1	0.33 U/μL
Taq DNA ligase (40 U/μL)	5	6.67 U/μL
Bst DNA polymerase (5 U/μL)	0.5	0.083 U/μL
**Total**	**15.5**	**N/A**

a In solution after step 2.b.
    ***Alternatives:*** When mapping abasic sites rather than oxidized guanine, replace EndoIV (1 μL) with T4 PNK (1 μL, 10 U/μL) and replace ThermoPol buffer (3 μL, 10×) with NEBuffer 2 (3 μL, 10×) at this stage.
  b. Add 15.5 μL of the reaction master mix to the prepared DNA to obtain a total volume of 30 μL.
  c. Mix the tube contents by flicking the side of each tube and spin the tubes briefly in a microcentrifuge.
  d. Incubate reactions at 37 °C for 60 min.
3. Purify the DNA using ProNex beads:
  a. Add 20 μL of ultrapure water and 80 μL of ProNex beads (sample:beads ratio 5:8) to each sample and mix well by flicking the tubes.
    ***Note:*** Equilibrate beads at 20–25 °C for 30 min and mix thoroughly by vortex before use.
  b. Incubate the samples at 20–25 °C for 10 min. (No shaking is required during this incubation.)
  c. Using a magnetic rack, remove supernatant from the beads and rinse the beads twice with 200 μL of ProNex Wash Buffer for 30-60 s while keeping the tube on the rack.
  d. Allow the samples to air-dry for 5 min.
  e. Elute the DNA from the beads by adding 25 μL of EB heated at 60 °C, carefully resuspend by flicking the tube and incubate for 10 min.
  f. Using a magnetic rack, transfer the eluted DNA into new tubes with minimal bead carryover.

### Enzymatic modification excision and propargyl-nucleotide incorporation

**Timing: 2 h**

This step excises target DNA modifications using corresponding enzymes and fills resulting gaps with propargyl-modified nucleotides, permanently encoding modification location within the DNA molecule.

4. Perform the excision reaction:
  a. Prepare a master mix containing the reagents below and mix well by pipetting.
    ***Note:*** Multiply the volumes by the number of reactions to be performed plus one in excess to ensure that the master mix suffices for all samples.

**Table:** 

Reagent	Volume for a single reaction (μL)	Final concentration^a^
NEBuffer 2 (10×)	3	1×
Fpg (8 U/μL)	1	8 U per sample
EndoIV (10 U/μL)	1	10 U per sample
**Total**	**5**	**N/A**

a In solution after step 4.b.
    ***Alternatives:*** When mapping abasic sites rather than oxidized guanine, replace Fpg (1 μL) with ultrapure water at this stage.
  b. Add 5 μL of the reaction master mix to the samples to obtain a total volume of 30 μL.
  c. Mix the tube contents by flicking the side of each tube and spin the tubes briefly in a microcentrifuge.
  d. Incubate reactions at 37 °C for 60 min.
    ***Note:*** No DNA purification is required before proceeding to the propargyl-nucleotide incorporation step.
5. Perform the propargyl-incorporation reaction:
  a. Prepare a master mix containing the reagents below and mix well by pipetting.
    ***Note:*** Multiply the volumes by the number of reactions to be performed plus one in excess to ensure that the master mix suffices for all samples.

**Table:** 

Reagent	Volume for a single reaction (μL)	Final concentration^a^
NEBuffer 2 (10×)	0.5	1×
Ultrapure water	0.8	N/A
Prop-dGTP (2.5 mM)	3.5	250 μM
Therminator IX (10 U/μL)	0.2	2 U per sample
**Total**	**5**	**N/A**

a In solution after step 5.b.
    ***Alternatives:*** When mapping abasic sites rather than oxidized guanine, replace the 3.5 μL of prop-dGTP (2.5 mM) with 1.75 μL of prop-dGTP (2.5 mM) and 1.75 μL of prop-dATP (2.5 mM).
  b. Add 5 μL of the reaction master mix to the samples to obtain a total volume of 35 μL.
  c. Mix the tube contents by flicking the side of each tube and spin the tubes briefly in a microcentrifuge.
  d. Incubate reactions at 60 °C for 10 min.
    ***Note:*** Samples may now be pipetted normally.
6. Purify the DNA using ProNex beads:
  a. Add 15 μL of ultrapure water and 80 μL of ProNex beads (sample:beads ratio 5:8) to each sample and mix well by pipetting.
    ***Note:*** Equilibrate beads at 20–25 °C for 30 min and mix thoroughly by vortex before use.
  b. Incubate the samples at 20–25 °C for 10 min.
  c. Using a magnetic rack, remove supernatant from the beads and rinse the beads twice with 200 μL of ProNex Wash Buffer for 30–60 s while keeping the tube on the rack.
  d. Allow the samples to air-dry for 5 min.
  e. Elute the DNA from the beads by adding 50 μL of EB heated at 60 °C, resuspend by pipetting and incubate for 10 min.
  f. Using a magnetic rack, transfer the eluted DNA with minimal bead carryover into new tubes suitable for use in QSonica Sonicator.
    **Pause point:** Samples can be stored at 4 °C for one day, otherwise at −20 °C.

### DNA fragmentation by sonication

**Timing: 1.5 h**

In this step, propargyl-labeled genomic DNA is fragmented by sonication to generate library-compatible fragment sizes. Fragmentation is performed after modification labeling to avoid introducing artifactual signals.

***Note:*** If enzymatic fragmentation is considered as an alternative to sonication, users should be aware that it may introduce sequence bias, potentially compromising genome-wide coverage uniformity and leaving certain genomic regions underrepresented.

7. Cool down Q800R2 Sonicator to 4 °C and sonicate for 5 min without any samples (20% amplitude) while the sonicator is cooling down.
8. Shear the DNA using the following parameters: 20% amplitude, pulse mode 15 s ON/5 s OFF, total 5 min, 4 °C.

**CRITICAL:** Adjust the water level using the corresponding knob. The water level should be exactly equal to the sample liquid inside the tubes.

***Note:*** The 5 min setting corresponds to cumulative ON time only. With a 15 s ON / 5 s OFF pulse program, the actual elapsed run time will exceed 5 min due to inclusion of OFF intervals.

***Note:*** These settings were optimized for use with a Q800R2 Sonicator and thin-wall individual PCR tubes (see Key Resources Table). When using a different sonicator, tube type, or DNA input other than mammalian genomic DNA, these settings should be re-optimized before starting the protocol.

**Figure 2:**
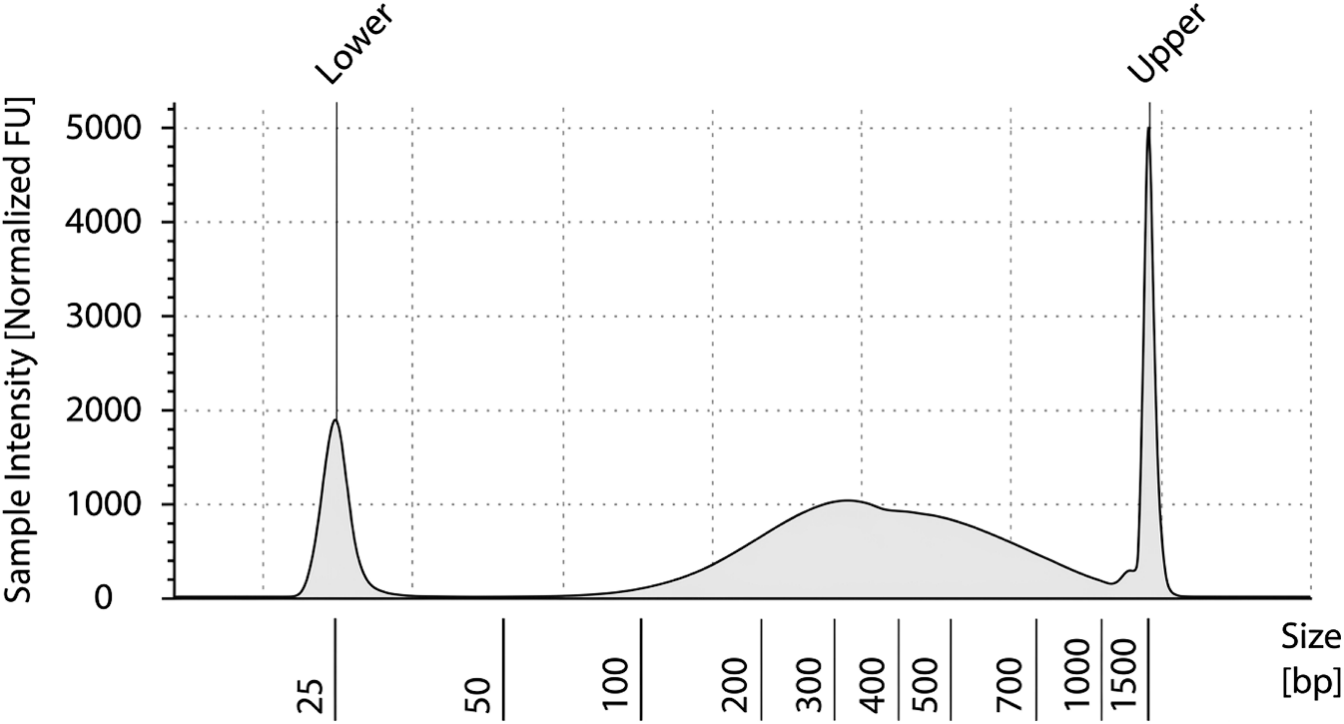
Representative electropherogram (TapeStation, D1000 ScreenTape) of genomic DNA after sonication Fragment size distribution peaks around 300–400 bp, consistent with optimal sonication conditions for library preparation. The Lower and Upper markers (25 bp and 1,500 bp, respectively) are indicated. Related to step 9.

***Note:*** The average fragment size should be around 400 bp.

9. Following the manufacturer’s instructions available here, check the size distribution of each sample using a capillary electrophoresis system (Agilent 4150 TapeStation) with D1000 screen tape. See Figure 2 for a representative electropherogram. troubleshooting 1.

10. Purify the DNA using ProNex beads:
  a. Add 100 μL of ProNex beads (sample:beads ratio 1:2, cutoff 150 bp) to each sample and mix well by pipetting.
    ***Note:*** Equilibrate beads at 20–25 °C for 30 min and mix thoroughly by vortex before use.
  b. Incubate the samples at 20–25 °C for 10 min.
  c. Using a magnetic rack, remove supernatant from the beads and rinse the beads twice with 200 μL of ProNex Wash Buffer for 30–60 s while keeping the tube on the rack.
  d. Allow the samples to air-dry for 5 min.
  e. Elute the DNA from the beads by adding 50 μL of EB (optionally preheated to 60 °C to improve elution efficiency), resuspend by pipetting and incubate for 5 min.
  f. Using a magnetic rack, transfer the eluted DNA into new tubes with minimal bead carryover.
11. Determine sample concentration using 1 μL of each sample with a fluorometric dsDNA quantification system (QuantiFluor ONE), following the manufacturer’s instructions available here.
12. Prepare 1 μg of fragmented DNA per sample in a final volume of 50 μL EB. troubleshooting 2.

**Pause point:** Samples can be stored at 4 °C for one day, otherwise at −20 °C.

### Adapter ligation with NEBNext Ultra II DNA Library Prep Kit

**Timing: 2.5 h**

In this step, genomic DNA fragments are first end-repaired and A-tailed, followed by ligation of custom P7 proximal sequencing adapters.

13. Perform an end polishing reaction:
  a. For each sample, mix the following components for a final volume of 60 μL.

**Table:** 

Reagent	Amount
NEBNext Ultra II End Prep Enzyme Mix	3 μL
NEBNext Ultra II End Prep Reaction Buffer	7 μL
Fragmented DNA (1 μg) from step 12	50 μL
  b. Set a 100 μL or 200 μL pipette to 50 μL and then pipette the entire volume up and down at least 10 times to mix thoroughly.
  c. Perform a quick spin to collect all liquid from the sides of the tube.
  d. Place in a thermocycler, with the heated lid set to 80 °C, and run the following program.

**Table:** 

Duration	Temperature
30 min	20 °C
30 min	65 °C
Hold	4 °C

14. Perform the ligation of the P7 proximal adapter:
  a. Add the following components to each sample for a total volume of 93.5 μL.

**Table:** 

Reagent	Amount
P7 proximal adapter mix (15 μM)	2.5 μL
NEBNext Ultra II Ligation Master Mix	30 μL
NEBNext Ligation Enhancer	1 μL
    **CRITICAL:** Mix the NEBNext Ultra II Ligation Master Mix by pipetting up and down several times prior to adding to the reaction.
  b. Set a 100 μL or 200 μL pipette to 50 μL and then pipette the entire volume up and down at least 10 times to mix thoroughly.
  c. Perform a quick spin to collect all liquid from the sides of the tube.
  d. Incubate at 20 °C for 15 min.
15. Purify the DNA using ProNex beads:
  a. Add 187 μL of ProNex beads (sample:beads ratio 1:2, cutoff 150 bp) to each sample and mix well by pipetting.
    ***Note:*** Equilibrate beads at 20–25 °C for 30 min and mix thoroughly by vortex before use.
  b. Incubate the samples at 20–25 °C for 10 min.
  c. Using a magnetic rack, remove supernatant from the beads and rinse the beads twice with 200 μL of ProNex Wash Buffer for 30–60 s while keeping the tube on the rack.
  d. Allow the samples to air-dry for 5 min.
  e. Elute the DNA from the beads by adding 50 μL of EB (optionally preheated to 60 °C to improve elution efficiency), resuspend by pipetting and incubate for 5 min.
  f. Using a magnetic rack, transfer the eluted DNA into new tubes with minimal bead carryover.
    **Pause point:** Samples can be stored at 4 °C for one day, otherwise at −20 °C.

### Click-chemistry-based MoDIS tagging

**Timing: 2.5 h**

This step covalently attaches the biotinylated MoDIS to propargyl-labeled modification sites via CuAAC, incorporating a validation code and a randomized index code that enable downstream filtering of amplification artifacts and deduplication of modification-supporting reads. The biotin moiety on MoDIS enables selective capture of modification-containing fragments by streptavidin-coated magnetic beads in the following step.

16. Concentrate samples to 6 μL using a vacuum concentrator at 35 °C.

**CRITICAL:** The next incubation must be performed in 0.2 mL PCR tubes/strips; ensure that the DNA is in the correct tube format.

***Note:*** If the vacuum concentrator accommodates 0.2 mL PCR tubes, concentrate directly in those. If only 1.5 mL tubes are compatible, concentrate in those and transfer to 0.2 mL PCR tubes. Briefly centrifuge before transfer to minimize losses.

17. Perform the click reaction to attach MoDIS:
  a. Prepare a master mix in a 0.5 mL tube containing the reagents below and mix well by pipetting.
    ***Note:*** Multiply the volumes by the number of reactions to be performed plus one in excess to ensure that the master mix suffices for all samples.
    **CRITICAL:** First add CuSO_4_ and THPTA and mix well by pipetting, the mixture should turn blue, confirming successful complexation of copper. Then add the remaining reagents and mix well.

**Table:** 

Reagent	Volume for a single reaction (μL)	Final concentration^a^
CuSO_4_ (20 mM)	1	1 mM
THPTA (200 mM)	1	10 mM
P5 proximal MoDIS Mix (250 μM)	4	50 μM
DMSO	4	20%
**Total**	**10**	**N/A**

a In solution after step 17.d.
  b. Incubate DNA samples from step 16 at 95 °C for 2 min.
  c. Spin down the samples and place on ice for at least 2 min.
  d. Add the following reagents to each sample in the order listed in the table below.

**Table:** 

Reagent	Amount
Master mix from step 17.a	10 μL
NaH_2_PO_4_ Buffer, pH 7 (1 M)	2 μL
  e. Gently mix samples by vortex and spin down.
  f. Add 2 μL of sodium ascorbate (400 mM) to each sample, mix by pipetting and immediately close the tube lid.
    **CRITICAL:** Prepare the sodium ascorbate solution (400 mM) on the day of the experiment and keep it on ice until use. To prepare it, dissolve 20 mg of (+)-sodium L-ascorbate in 250 µL of ultrapure water.
    **CRITICAL:** Add sodium ascorbate individually to each sample (or to each strip if using a multichannel pipette) to enable immediate closure of the tube lid.
  g. Incubate samples at 37 °C for 60 min.
18. Purify the DNA using ProNex beads:
  a. Add 30 μL of water and 100 μL of ProNex beads (sample:beads ratio 1:2, cutoff 150 bp) to each sample and mix well by pipetting.
    ***Note:*** Equilibrate beads at 20–25 °C for 30 min and mix thoroughly by vortex before use.
  b. Incubate the samples at 20–25 °C for 10 min.
  c. Using a magnetic rack, remove supernatant from the beads and rinse the beads twice with 200 μL ProNex Wash Buffer for 30–60 s while keeping the tube on the rack.
  d. Allow the samples to air-dry for 5 min.
  e. Elute the DNA from the beads by adding 30 μL of EB (optionally preheated to 60 °C to improve elution efficiency), resuspend by pipetting and incubate for 5 min.
  f. Using a magnetic rack, transfer the eluted DNA into new tubes with minimal bead carryover.
    **Pause point:** Samples can be stored at 4 °C for one day, otherwise at −20 °C.

### Biotin-streptavidin enrichment of MoDIS-tagged fragments

**Timing: 1 h**

This step selectively captures MoDIS-tagged DNA fragments using streptavidin-coated magnetic beads, removing unlabeled genomic fragments and substantially increasing the proportion of sequencing reads derived from genuine modification-containing molecules.

***Note:*** Non-specific binding during streptavidin pulldown may occur; however, specificity is primarily ensured at the computational level through validation code filtering, which removes reads not containing a valid MoDIS sequence. The fraction of reads passing MoDIS barcode filtering reported in the preprocessing summary plot serves as the key quality control metric for this step.

19. Mix thoroughly by vortex Dynabeads MyOne Streptavidin C1 (streptavidin beads) for 30 s.
20. Take 10 μL of streptavidin beads per sample into a single tube (e.g., 80 µL for eight samples), place on magnetic stand and discard supernatant. Steps 21–24 are performed on this pooled bead suspension, which is divided into individual tubes at step 25.
21. Add 1 mL of 1x Bind and Wash buffer, mix thoroughly by vortex, place on magnetic stand and discard supernatant.
22. Add 300 μL of 1× Bind and Wash buffer, mix thoroughly by vortex, place on magnetic stand and discard supernatant.
23. Repeat step 22.
24. Resuspend washed streptavidin beads from step 23 in 30 μL of 2× Bind and Wash buffer per sample (e.g., 240 µL for eight samples).
25. Transfer 30 μL of streptavidin beads from step 24 into one new empty tube per sample.
26. Incubate DNA samples from step 18.f at 95 °C for 2 min.
27. Spin down the samples and place on ice for at least 2 min.
28. Transfer 30 μL DNA samples to the tubes containing the streptavidin beads from step 25.
29. Incubate for 15–30 min at 20–25 °C with gentle rotation of the tube.
30. Spin down, place on magnetic rack for 1 min and discard supernatant.
31. Add 200 μL of 1× Bind and Wash buffer to each tube, mix thoroughly by vortex, rotate gently for 3 min, spin down, place on magnetic rack for 1 min and discard supernatant.
32. Repeat step 31 twice, for a total of three washes.
33. Add 200 μL of EB to each tube, mix thoroughly by vortex, rotate gently for 3 min, spin down, place on magnetic rack for 1 min and discard supernatant.
34. Resuspend in 20 μL of EB and transfer to 0.2 mL PCR tubes or strips.

### Initial amplification with triazole bypass

**Timing: 1.5 h**

This step amplifies enriched modification-containing fragments using Vent (exo-) DNA polymerase, which efficiently bypasses the triazole linkage introduced by the click reaction. Newly synthesized, linkage-free DNA is recovered during the ProNex beads purification, while fragments still containing the triazole linkage remain bound to the streptavidin beads and are discarded with the beads.

35. Perform the triazole-bypass initial amplification:
  a. Prepare a master mix containing the reagents below and mix well by pipetting.
    ***Note:*** Multiply the volumes by the number of reactions to be performed plus one in excess to ensure that the master mix suffices for all samples.

**Table:** 

Reagent	Volume for a single reaction (μL)	Final concentration^a^
Ultrapure water	16.5	N/A
ThermoPol buffer (10×)	5	1×
dNTPs (2 mM)	5	200 μM
MgSO4 (100 mM)	1	2 mM
P5_prox_primer (10 μM)	1	0.2 μM
P7_prox_primer (10 μM)	1	0.2 μM
Vent (exo-)DNA Polymerase (2 U/μL)	0.5	0.02 U/μL
**Total**	**30**	**N/A**

a In solution after step 35.b.
  b. Add 30 μL of the reaction master mix to the samples from step 34 to obtain a total volume of 50 μL.
  c. Mix the tube contents by pipetting and spin the tubes briefly in a microcentrifuge.
  d. Place in a thermocycler and run the following PCR program.

**Table:** 

Steps	Temperature	Time	Cycles
Initial Denaturation	95 °C	120 s	1
Denaturation	95 °C	20 s	5 cycles
Annealing	59 °C	20 s	
Extension	72 °C	60 s	
Final extension	72 °C	300 s	1
Hold	4 °C	infinite

36. Purify the DNA using ProNex beads:
  a. Add 100 μL of ProNex beads (sample:beads ratio 1:2, cutoff 150 bp) to each sample and mix well by pipetting.
    ***Note:*** Equilibrate beads at 20–25 °C for 30 min and mix thoroughly by vortex before use.
  b. Incubate the samples at 20–25 °C for 10 min.
  c. Using a magnetic rack, remove supernatant from the beads and rinse the beads twice with 200 μL ProNex Wash Buffer for 30–60 s while keeping the tube on the rack.
  d. Allow the samples to air-dry for 5 min.
  e. Elute the DNA from the beads by adding 45 μL of EB (optionally preheated to 60 °C to improve elution efficiency), resuspend by pipetting and incubate for 5 min.
  f. Using a magnetic rack, transfer the eluted DNA into new tubes with minimal bead carryover.
    **Pause point:** Samples can be stored at 4 °C for one day, otherwise at −20 °C.

### Indexing and amplification

**Timing: 1.5 h**

This step amplifies recovered DNA fragments using dual-indexing primers to generate sequencing-ready libraries.

37. Amplify the library with the following PCR:
  a. Mix the following components in 0.2 mL PCR tubes or strips, one for each sample from step 36.

**Table:** 

Reagent	Amount
NEBNext Ultra II Q5 Master Mix	50 μL
P5 Index Primer (20 μM) [i5_N]	5 μL
P7 Index Primer (20 μM) [i7_N]	5 μL
DNA from step 36.f	40 μL
    ***Note:*** Assign one unique combination of i7 and i5 primers to each library, where N denotes the index number supplied in the primer set.
  b. Place in a thermocycler and run the following PCR program.

**Table:** 

Steps	Temperature	Time	Cycles
Initial Denaturation	95 °C	120 s	1
Denaturation	95 °C	20 s	10–15
Annealing	59 °C	20 s	
Extension	72 °C	60 s	
Final extension	72 °C	300 s	1
Hold	4 °C	infinite

***Note:*** The necessary number of cycles varies depending on the yield of each sample and typically ranges from 10 to 15 cycles. 13 cycles is a recommended starting point. The optimal number can be estimated by qPCR using 2 μL of sample and any indexing primers prior to the amplification step: plot the fluorescence signal versus cycle number and use the cycle corresponding to 1/4 of the maximum fluorescence.^20^ The optimal threshold may need to be adjusted depending on sample type, instrument, and laboratory setup. Alternatively, the optimal cycle number can be empirically determined by first amplifying a single test sample, assessing library quality and concentration, and adjusting the number of cycles for the remaining samples accordingly.

38. Perform a double-sided purification of the DNA using ProNex beads:
  a. Add 100 μL of ProNex beads (sample:beads ratio 1:1, upper cutoff 1000 bp) to each sample and mix well by pipetting.
    ***Note:*** Equilibrate beads at 20–25 °C for 30 min and mix thoroughly by vortex before use.
  b. Incubate the samples at 20–25 °C for 10 min.
  c. Using a magnetic rack, carefully transfer supernatant into a new tube containing 35 μL of ProNex beads (cumulative sample:beads ratio 1.35:1, lower cutoff ∼320 bp) and mix well by pipetting.
    **CRITICAL:** The sample is in the supernatant, do not discard.
  d. Incubate the samples at 20–25 °C for 10 min.
  e. Using a magnetic rack, remove supernatant from the beads and rinse the beads twice with 200 μL of ProNex Wash Buffer for 30–60 s while keeping the tube on the rack.
  f. Allow the samples to air-dry for 5 min.
  g. Elute the DNA from the beads by adding 30 μL of EB (optionally preheated to 60 °C to improve elution efficiency), resuspend by pipetting and incubate for 5 min.
  h. Using a magnetic rack, transfer the eluted DNA into new tubes with minimal bead carryover.

### Quality control and pooling

**Timing: 1.5 h**

This step assesses the concentration and size distribution of each indexed library before pooling at equimolar ratios for high-throughput sequencing.

39. Check the DNA concentration using 1 μL of the library from step 38.h with a fluorometric dsDNA quantification system (QuantiFluor ONE), following the manufacturer’s instructions available here.

***Note:*** A DNA yield of 0.5–4 ng/μL is expected. troubleshooting 3.

***Note:*** If the DNA concentration is < 0.5 ng/μL, achieving the final 15 nM pool may not be possible. While re-amplification can be performed, it is better to use additional PCR cycles at step 37.b. Re-amplification increases PCR-induced biases and requires an additional purification step, adding time, cost and potential variability.

**Figure 3:**
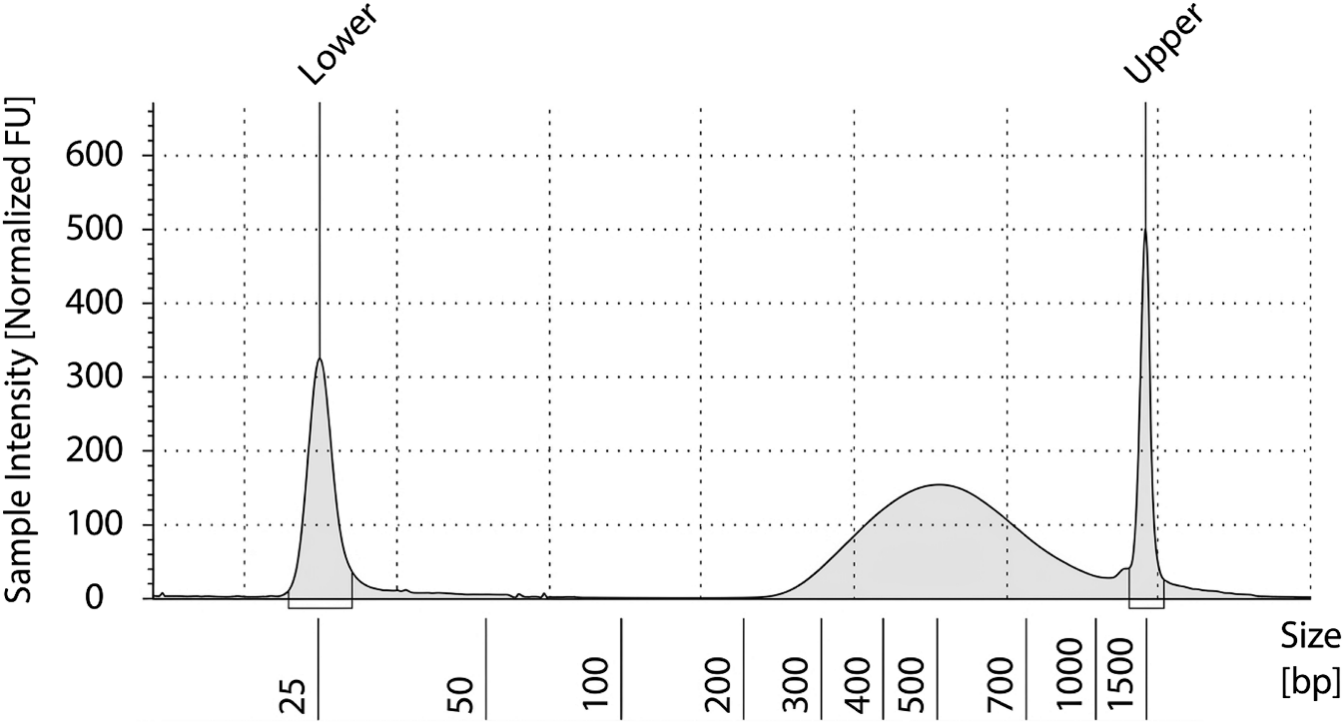
Representative electropherogram (TapeStation, HS D1000 ScreenTape) of a click-code-seq library after final indexing and size selection The library shows a broad size distribution enriched between 250 and 1,000 bp with no prominent adapter-dimer peak below 150 bp, indicative of a high-quality library. The Lower and Upper markers (25 bp and 1,500 bp, respectively) are indicated. Related to step 40.

40. Following the manufacturer’s instructions available here, check the size distribution of each library from step 38.h using a capillary electrophoresis system (Agilent 4150 TapeStation) with HS D1000 screen tape. See Figure 3 for a representative electropherogram.

**CRITICAL:** A good DNA library contains no adapter/primer dimer peaks below 150–180 bp, as primer dimers contribute to invalid sequencing reads. Libraries to be pooled should have comparable size distributions, predominantly between 400 and 600 bp, with only a minor fraction of fragments above 1,000 bp, as molecules of different sizes have different clustering efficiency during sequencing. If your samples do not meet these criteria, return to step 38 for an additional round of purification. If all samples meet these criteria, record the average fragment size of each library and proceed to the next step.

41. Calculate the molar concentration of each library as follows: Molar concentration (nM) = [concentration (ng/μL) / (660 (g/mol/bp) × average fragment size (bp))] × 10^6^.
42. Pool the libraries in equimolar amounts to reach the specifications of your sequencing facility.

***Note:*** Consult your sequencer operator regarding minimal sample concentration and volume.

43. Perform a ProNex clean up with a cutoff at 250 bp following the manufacturer’s instructions available here and elute in a proper volume to reach specifications of your sequencer facility.
44. Quality control of the pool:
  a. Determine the DNA concentration using 1 μL of the pool with a fluorometric dsDNA quantification system (QuantiFluor ONE), following the manufacturer’s instructions available here.
  b. Following the manufacturer’s instructions available here, check the size distribution of the pool using a capillary electrophoresis system (Agilent 4150 TapeStation) with HS D1000 screen tape to determine the average fragment size of the pool.
45. Sequence the pooled libraries on an Illumina NovaSeq X or equivalent Illumina-compatible short-read sequencing instrument with 150-bp paired-end reads. Aim for 40 million reads per library.

### Data analysis

**Timing:** Variable (typically up to 1 day for human genome samples, depending on the computing cluster and sequencing depth; ∼10 min for the test dataset provided on GitLab). Hands-on time is approximately 1–2 h on first use, including conda environment creation, pipeline configuration, and job submission. For subsequent runs, hands-on time is less than 30 min and is limited to configuration and job submission.

This section describes the preprocessing of raw sequencing data into genome-aligned BED files, which serve as the basis for all downstream genomic and bioinformatic analyses. The preprocessing pipeline Click-pipe performs: adapter trimming, MoDIS barcode filtering, UMI-guided deduplication, and alignment to produce single-nucleotide-resolution DNA modification maps. Downstream analyses are not described here but example scripts are available at https://gitlab.ethz.ch/eth_toxlab/click-code-seq, accompanying the associated publication.^1^

***Note:*** This protocol uses an automated, SLURM-based preprocessing pipeline designed for high performance computing environments. Although developed on the Euler cluster at ETH Zurich, the pipeline can be adapted to other SLURM-based or alternative scheduling systems with appropriate modifications to job submission scripts. troubleshooting 4.

46. Install the pipeline:
  a. Clone the pipeline into your project directory using git:
    ***Note:*** Alternatively, download the latest release from the GitLab page here: https://gitlab.ethz.ch/eth_toxlab/click_pipe
    git clone https://gitlab.ethz.ch/eth_toxlab/click_pipe.git
    cd click_pipe
  b. Create and activate the conda environment:

conda env create -f ClickPipeEnv.yml -n clickpipe

conda activate clickpipe

***Note:*** All required software is managed through the clickpipe conda environment. No additional software installation is required.

47. Download the reference genome:
  a. For human DNA, download the pre-built GRCh38 (hg38) Bowtie2 index and extract the corresponding FASTA file:
    wget https://genome-idx.s3.amazonaws.com/bt/GRCh38_noalt_as.zip
    unzip GRCh38_noalt_as.zip
    bowtie2-inspect /path/to/GRCh38_noalt_as/GRCh38_noalt_as > GRCh38_noalt_as.fasta
  b. For mouse DNA, download the pre-built GRCm39 (mm39) index and extract the FASTA file:

wget https://genome-idx.s3.amazonaws.com/bt/GRCm39.zip

unzip GRCm39.zip

bowtie2-inspect /path/to/GRCm39/GRCm39 > GRCm39.fasta

***Note:*** For other organisms, download or generate a Bowtie2 index from the reference genome FASTA file following the Bowtie2 documentation.

48. Configure the pipeline by editing config.json to reflect your file system and reference genome, using the template below:

{

"raw_file_folder": "/path/to/raw_fastq_files/",

"scratch_folder": "/path/to/scratch/working_directory/",

"output_folder": "/path/to/permanent/output_directory/",

"reference_genome": "/path/to/bowtie2_index/genome_basename",

"genome_fasta": "/path/to/genome.fasta",

"target_nucleotide": "G"

}

***Note:*** The paths are defined as follows: raw_file_folder is the directory containing the raw.fastq.gz files (both R1 and R2 required); scratch_folder is the working directory where intermediate files are written; output_folder is the directory for long-term storage of completed results; reference_genome is the Bowtie2 index basename (file path before .1.bt2); genome_fasta is the reference genome FASTA file; and target_nucleotide is G when mapping oxidative DNA modification, AG when mapping AP sites.

49. Launch the pipeline running cells 1, 2, and 3 of the Jupyter notebook click_pipe.ipynb. Troubleshooting 5, 6, 7, and 8.

***Note:*** If JupyterHub is not available on your cluster, a JupyterLab session can be started manually using an SSH tunnel, see the Click-pipe README for detailed instructions.

***Alternatives:*** Launch the pipeline with the following command:

./run_click_pipe.sh <WORKING_PATH> <OUTPUT_PATH>

Where: WORKING_PATH is the working directory where intermediate files are written, and OUTPUT_PATH is the directory for long-term storage of completed results.

***Note:*** All steps are submitted as SLURM jobs with automatically assigned dependencies, ensuring that each step starts only once all jobs from the preceding step are completed successfully.

***Note:*** The automated preprocessing pipeline consists of seven sequential steps. **Step 0** performs initial quality control on the raw FASTQ files. **Step 1** removes adapters and low-quality bases (Phred score < 20, minimum read length 50 bp) from both reads using Trimmomatic, crops the nongenomic sequence from Read 2, and filters read pairs based on MoDIS barcode recognition while keeping R1/R2 synchronized. **Step 2** extracts VC and RIC from the reads and appends them to the read names to enable downstream UMI-guided deduplication. **Step 3** aligns the processed reads to the reference genome using Bowtie2 and produces coordinate-sorted alignment files. **Step 4** sorts the BAM files, retains only properly paired alignments, removes unmapped or unpaired reads, and generates postfiltering alignment statistics. **Step 5** collapses PCR duplicates using the RIC information to generate a deduplicated BAM for each sample, using the *--method=cluster* option of UMI-tools dedup. **Step 6** keeps the forward mate only, converts the deduplicated BAM to BED format, computes strand corrected modification coordinates, removes entries with negative genomic starts or subthreshold mapping quality (MAPQ < 20), and extracts the 1, 3, and 21 nt sequence context windows from the reference genome to produce the final BED files used for downstream analyses. After completion of Steps 1, 4, 5, and 6, auxiliary scripts are automatically executed to record the number of reads retained at each stage. These statistics are stored in a JSON file named *all_stats.json*, which is used after Step 6 to generate the preprocessing summary plot.

50. Once the pipeline has completed, open a fresh session in click_pipe.ipynb, run Cell 1 and Cell 2 (skip Cell 3), then run any post-processing cell independently to generate consolidated QC reports (MultiQC), collect final BED files, split BED files by strand, and gather SLURM logs.

***Note:*** If running via JupyterHub, MultiQC must be installed separately before running the post-processing cells: pip install multiqc. If running via a manually started JupyterLab session using the clickpipe conda environment, MultiQC is already included and no additional installation is needed.

***Note:*** A companion notebook, genome_wide_view.ipynb, is also provided for a first look at genome-wide DNA modification levels. It bins modification counts into genomic windows, normalizes by nucleotide content and sequencing depth, and produces chromosome-by-chromosome plots for each sample.

## Expected outcomes

During preprocessing, intermediate step folders are written to scratch_folder and automatically copied to output_folder as each step completes. Once all steps have finished and post-processing has been run, the final BED files for each sample are stored in:

/output_folder/bed_files/1nt/sampleName_MAPQ20_seq_1nt.bed

/output_folder/bed_files/3nt/sampleName_MAPQ20_seq_3nt.bed

/output_folder/bed_files/21nt/sampleName_MAPQ20_seq_21nt.bed

For each sample, the pipeline generates three final BED files, corresponding to three different sequence-context windows extracted around each mapped modification site. The 1nt BED file contains only the modified nucleotide and is used for base-level localization and strand-bias analyses. The 3nt BED file contains a trinucleotide window centered on the modification and is used for trinucleotide-context profiling. The 21 nt BED file contains an extended 21-base window surrounding the site and is used for broader sequence-context analyses and motif discovery.

Each line in the BED files contains: the chromosome, the 0-based start coordinate of the interval, the end coordinate (exclusive), the identifier of the sequencing read supporting the call, the Bowtie2 mapping-quality score (MAPQ), the strand on which the read aligned, and the extracted sequence context (1, 3 or 21 nt) surrounding the mapped DNA-modification site. Representative entries for each resolution are shown below:

# 1 nt resolution

chr1 16335 16336 <readID> 30 + G

# 3 nt resolution

chr1 16334 16337 <readID> 30 + AGT

# 21 nt resolution

chr1 16325 16346 <readID> 30 + TGTTGCTGTAGTTTGTTATTA

These BED files form the starting point for downstream analyses, including genomic-feature annotation, sequence-context profiling, and the computation of transcriptional or replicative strand biases. Example analysis scripts are available at https://gitlab.ethz.ch/eth_toxlab/click-code-seq, accompanying the associated publication.^1^

**Figure 4:**
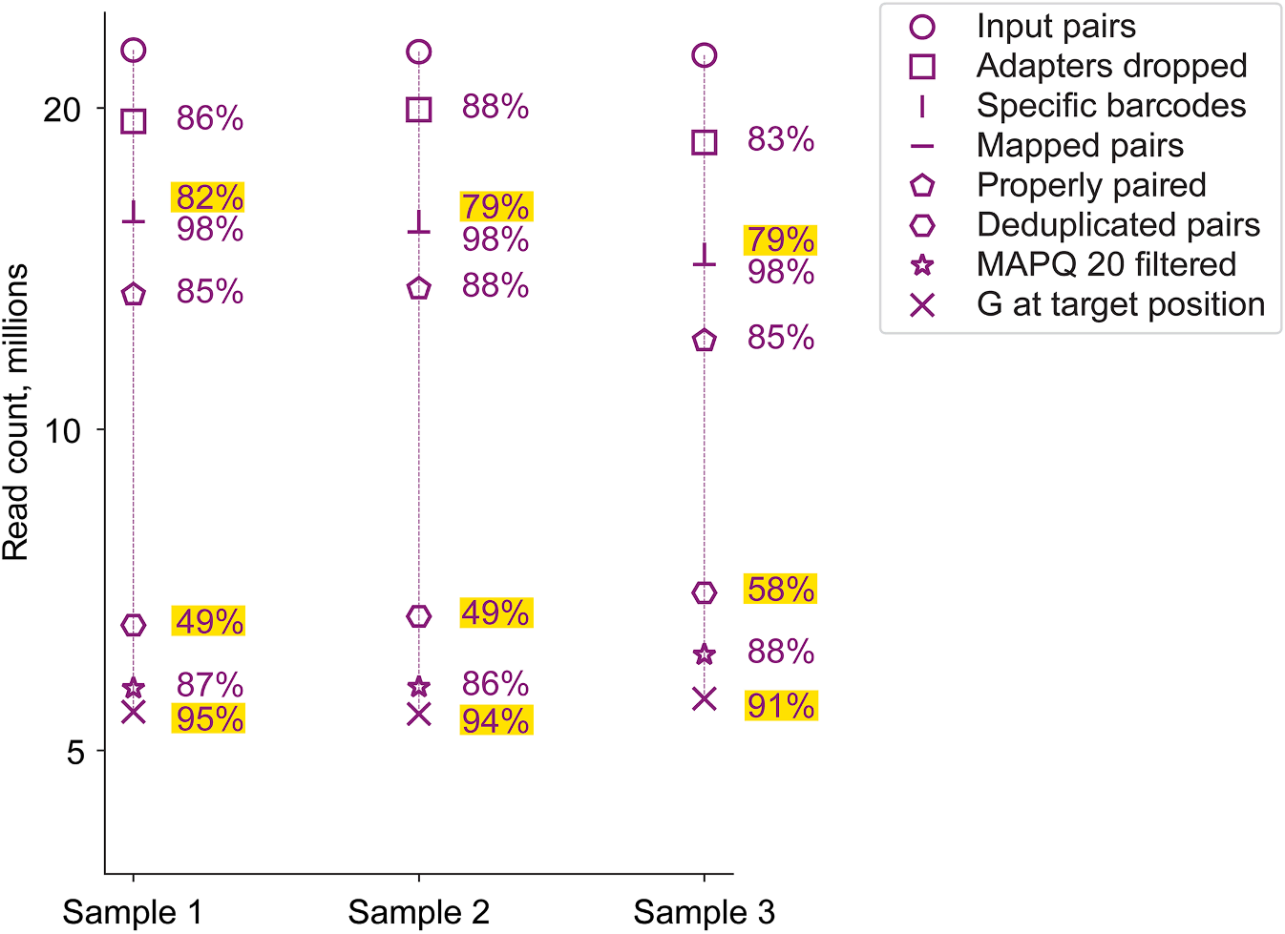
Representative preprocessing summary plot for three click-code-seq libraries Each symbol indicates the number of read pairs retained after successive preprocessing steps: raw input pairs (○), pairs retained after adapter trimming (□), pairs passing MoDIS barcode filtering (|), pairs successfully aligned to the reference genome (−), properly paired alignments (⬠), pairs remaining after UMI-guided deduplication (⬡), reads retained after mapping quality filtering (MAPQ ≥ 20) (☆), and reads with the expected nucleotide at the called modification position (×). Read counts are displayed on a logarithmic scale. Percentages indicate the fraction of reads retained relative to the preceding step. Highlighted values indicate the key quality control metrics used to assess library performance. Related to step 49.

In addition to the final BED files, the pipeline generates a preprocessing summary plot named preprocessing_stats_plotted.png (see Figure 4 for a representative example), which is saved in the directory specified by output_folder. This plot summarizes read retention across key preprocessing steps for all processed samples and provides a compact overview of data loss and filtering efficiency throughout the pipeline. In the plot, each sample is shown along the x axis and read counts are displayed on a logarithmic scale on the y axis. Symbols indicate the number of read pairs retained after successive preprocessing steps: ***Input pairs*** correspond to raw read pairs entering the pipeline; ***Adapters dropped*** indicate read pairs retained after adapter and quality trimming; ***Specific barcodes*** represent read pairs passing MoDIS barcode filtering; ***Mapped pairs*** indicate read pairs successfully aligned to the reference genome; ***Properly paired*** indicates read pairs with correct pairing and orientation; ***Deduplicated pairs*** represent read pairs remaining after UMI-guided deduplication; ***MAPQ 20 filtered*** indicates reads retained after mapping quality filtering; and ***G at target position*** (or G/A at target position, depending on the target nucleotide defined in the configuration file) indicates the number of retained reads whose called nucleotide corresponds to the expected base for the mapped DNA modification.

Percentages shown next to each symbol indicate the fraction of reads retained relative to the preceding step, enabling rapid identification of stages at which read loss occurs. This plot is intended as a quality control overview to assess preprocessing performance across samples and to help identify deviations that may require further inspection using the troubleshooting section.

## Quantification and statistical analysis

The data obtained requires normalization to account for differences in target-nucleotide content across genomic features and for variation in sequencing depth between samples.

First, DNA-modification counts are normalized to the abundance of the target nucleotide in the reference genome. For each genomic feature *f*, the raw number of mapped DNA-modification events *N(s,T,f)* in sample *s* and modification type *T* is divided by the number of target nucleotides for *T* within that feature, denoted *N_ref_(T,f)*. Target nucleotides correspond to guanine for oxidized guanine and to adenine or guanine for deoxyadenosine-derived or deoxyguanosine-derived AP sites, respectively. This normalization corrects for sequence composition biases across genomic features.

Second, to correct for differences in sequencing depth and overall modification load between samples, a sample-specific normalization factor is computed using genome-wide 100-kb bins. For each sample *s* and modification type *T*, the normalization factor *M(s,T)* is defined as the median across all 100-kb genomic bins *b* of the nucleotide-normalized modification density:

$$M\left(s,T\right)={median}_{across\;b}{\left\{\frac{N\left(s,T,b\right)}{N_{ref}\left(T,b\right)}\right\}}_{b}$$

Here, *N(s,T,b)* is the number of mapped DNA-modification events of type *T* within bin *b* in sample *s*, and *N_ref_(T,b)* is the number of target nucleotides for *T* within the same bin in the reference genome, with both DNA strands considered together. Using the median across large genomic bins provides a robust, hotspot-insensitive estimate of genome-wide sequencing depth, which is used here as a normalization factor. This bin size is independent of the resolution at which modifications are subsequently analyzed; downstream analyses can be performed at any resolution, including single-nucleotide, gene-level, or user-defined bin sizes.

Final normalized DNA-modification levels *C(s,T,f)* for any genomic feature f are calculated as:

$$C\left(s,T,f\right)=\frac{N\left(s,T,f\right)}{N_{ref}\left(T,f\right)\cdot M\left(s,T\right)}$$

Normalized values are reported in arbitrary units. This normalization framework is applied consistently across different feature types, including dinucleotides, fixed-size chromosome bins, gene strands, transcription start site-adjacent regions, and other user-defined genomic annotations. There can also be alternative approaches to compute the sample-specific normalization factors, for example, using the gene bodies of unexpressed genes instead of chromosome bins (see more details in the original work^1^).

## Limitations

Click-code-seq maps the substrate scope of the excision enzyme rather than directly measuring a specific modification structure. For example, Fpg excises multiple oxidized guanine substrates, including 8-oxoG and formamidopyrimidines, so the resulting map reflects all Fpg substrates rather than a single defined modification. Click-code-seq likely underestimates closely spaced modifications occurring within approximately 150 bp on the same DNA strand due to size selection during library preparation, resulting in detection of only the 5′-proximal modification. While modification clusters present across many cells remain detectable, clusters confined to individual DNA molecules may be underrepresented. Consistent with the exceedingly low density of such chemical modifications in the genome, the protocol requires substantial sequencing depth and computational analysis, contributing to higher cost and analytical complexity compared with either standard DNA sequencing or location-independent quantification of modifications. We recommend a minimum of 40 million reads per library, though the optimal depth will depend on the abundance of the target modification and the complexity of the genome being studied. Oxidative DNA modifications can be introduced artifactually during DNA extraction and handling, potentially contributing to false-positive signals; the use of antioxidants during DNA extraction, such as PBN (α-phenyl-N-tert-butylnitrone), can help minimize artifactual oxidation, though it is difficult to verify effectiveness for particular samples. As with other omics-based approaches, click-code-seq may be subject to batch effects arising from differences in reagent lots, library preparation timing, or sequencing runs, the extent and impact of which have not yet been fully characterized and warrant further investigation.

## Troubleshooting

### Problem 1

The average fragment size after sonication is below 300 bp or above 600 bp (related to step 9), most commonly due to suboptimal sonication parameters, which vary with sonicator model, tube type, DNA input amount, and sample volume.

### Potential solution

• Optimize sonication settings (amplitude, pulse duration, total time) for your specific sonicator, tube type, and DNA input before beginning the full protocol.
• Ensure the water level in the sonicator bath is exactly equal to the sample liquid level inside the tubes to reduce splashing and subsequent uneven sonication.
• For fragments below 300 bp, reduce sonication time or amplitude.
• For fragments above 600 bp, increase sonication time and re-analyze fragment size distribution before proceeding.

### Problem 2

The DNA amount falls below 1 μg after ProNex bead clean-up (related to step 12), most commonly due to suboptimal bead equilibration, incomplete elution, or excessive pipetting of high-molecular-weight genomic DNA.

### Potential solution

• Ensure ProNex beads are equilibrated at 20–25 °C for at least 30 min and mixed thoroughly by vortex before use to obtain a homogeneous suspension.
• Extend elution time to 15 min and ensure EB is heated to 60 °C.
• Consider increasing DNA input to 4 μg to anticipate losses.
• If no additional material is available, proceed with all recovered DNA.
• If DNA concentration was measured by absorbance (e.g., NanoDrop), RNA contamination may lead to a significant overestimation of DNA concentration, resulting in insufficient DNA input in subsequent steps. We recommend quantifying DNA using a fluorometric method with a DNA-intercalating dye (e.g., Quantus fluorometer with QuantiFluor ONE dsDNA System, or a Qubit fluorometer with dsDNA assay), which is specific to double-stranded DNA and insensitive to RNA contamination. If a NanoDrop measurement was used, check the 260/280 ratio: a ratio significantly above 1.8 may indicate RNA contamination, in which case RNA removal with RNase A treatment followed by re-quantification may be considered, although this has not been specifically tested in the context of this protocol.

### Problem 3

More than 25 PCR cycles are required for final library amplification, or the final library concentration is higher than expected (related to step 39), indicating either low library complexity resulting from insufficient input DNA or low abundance of target modifications, or overamplification resulting in high duplication rates and reduced data quality.

### Potential solution

• If more than 25 cycles are required, proceed with sequencing regardless of the cause. Evaluate the preprocessing statistics to determine whether low yield reflects low modification abundance (sufficient unique reads with good MoDIS specificity) or a library preparation failure (very low MoDIS specificity).
• If the preprocessing statistics indicate library preparation failure, verify the specifications of the prop-dGTP and MoDIS syntheses, confirm modification excision and propargyl-nucleotide incorporation efficiency using the click-fluoro-quant assay described in Takhaveev et al^1^, and repeat library preparation with increased starting DNA input if material is available.
• It is better to use more PCR cycles at the indexing step than to re-amplify after purification, as re-amplification increases PCR bias and requires an additional purification step.
• If overamplification has occurred (library concentration higher than expected), proceed with sequencing and allocate additional reads to compensate for increased duplication rates.

### Problem 4

The preprocessing pipeline may fail or produce errors on computing clusters other than the Euler cluster at ETH Zurich (related to step 46), as the pipeline submission scripts contain cluster-specific SBATCH directives that may not be compatible with other high-performance computing environments.

### Potential solution

• SLURM directives: Edit the job-submission headers in all pipeline scripts (Step 0 through Step 6 and run_click_pipe.sh) to match the scheduler syntax, queue structure, and resource specifications of your cluster. Parameters that typically require adjustment include --time, --cpus-per-task, --mem-per-cpu, and output/error file paths.
• Non-SLURM schedulers: If your cluster uses a different job scheduler, translate all SBATCH directives into the appropriate submission syntax for your system and update the job-dependency logic accordingly.
• Verification: Before processing a full dataset, we recommend first running the test dataset provided in test_data/ to confirm that the scheduler accepts the resource settings, that the clickpipe conda environment activates correctly, and that job dependencies execute in the intended order.

### Problem 5

The fraction of G at called sites is close to 25% rather than the expected >80% when mapping oxidized guanines with Fpg and prop-dGTP (related to step 49), indicating that the click reaction or upstream labeling steps have failed, resulting in non-specific library enrichment rather than genuine modification detection.

### Potential solution

• Check the specifications of the prop-dGTP and MoDIS syntheses to confirm correct chemical modification.
• Ensure sodium ascorbate is prepared fresh on the day of the experiment. Oxidized sodium ascorbate is a common cause of click reaction failure.
• Verify enzyme storage conditions and shelf life for Fpg, EndoIV, and Therminator IX.
• Confirm that EB does not contain EDTA which inhibits the metal-dependent enzymes used in this protocol.

### Problem 6

MoDIS specificity falls below 75% after data processing (related to step 49), indicating either an inefficient click reaction or inefficient biotin enrichment, resulting in a high proportion of reads that do not derive from genuine modification-containing fragments.

### Potential solution

• For click reaction failure, see troubleshooting solutions in Problem 5 above.
• For inefficient biotin enrichment, ensure thorough washing steps during streptavidin bead capture and transfer beads to fresh tubes after the first wash.

### Problem 7

The alignment rate falls below 85% after data processing (related to step 49), typically indicating sample or reagent contamination, or use of an incorrect reference genome.

### Potential solution

• Confirm that the correct reference genome and Bowtie2 index are specified in the pipeline configuration file.
• If contamination is suspected, identify the source by aligning a subset of unmapped reads to potential contaminant genomes (e.g., bacterial, mycoplasma). If contamination cannot be resolved, replace all reagents and repeat library preparation.

### Problem 8

The deduplication rate is high after UMI-guided deduplication (related to step 49), indicating library overamplification resulting in many PCR duplicates that are collapsed during deduplication and reducing the number of unique modification-supporting reads.

### Potential solution

• If more than 2 million unique reads are obtained after deduplication, proceed with data analysis as the library complexity is sufficient.
• If fewer than 2 million unique reads are obtained, resequencing the library can be considered to increase unique read depth.
• For future preparations, use fewer PCR cycles at the indexing step to reduce duplication rates.

## Resource availability

### Lead contact

Further information and requests for resources and reagents should be directed to and will be fulfilled by the lead contact, Shana J. Sturla (sturlas@ethz.ch).

### Technical contact

Technical questions on executing this protocol, including the computational pipeline, should be directed to and will be answered by the technical contact, Vakil Takhaveev (vtakhaveev@ethz.ch).

### Materials availability

The custom-synthesized oligonucleotides used in this protocol, including prop-dGTP, prop-dATP, MoDIS_1, MoDIS_2, and P7_prox adapters, can be obtained from commercial suppliers as described in the materials and equipment section. The design of MoDIS and its use in click-code-seq are covered by patent application PCT/EP2024/068922 (WO2025012092A1), filed by ETH Zurich. Researchers wishing to use MoDIS for commercial purposes should contact ETH Zurich technology transfer. All other materials used in this study are commercially available as listed in the key resources table.

### Data and code availability

The click-code-seq preprocessing pipeline Click-pipe is publicly available at https://gitlab.ethz.ch/eth_toxlab/click_pipe and archived on Zenodo: https://doi.org/10.5281/zenodo.20327994.

## Acknowledgments

The project was supported by a Longevity Impetus Grant from Norn Group, Hevolution Foundation and Rosenkranz Foundation. The representative data used in this paper were generated using the resources of the ETH Zurich Genetic Diversity Center and the ETH Zurich Euler cluster. We thank J. Kubitschek (ETH Zurich) for discussions on click-code-seq methodology and support in the development of Click-pipe, and L.L.M. Derks (ETH Zurich) for testing and providing feedback on Click-pipe.

## Author contributions

L.L. wrote the manuscript with contributions from all other authors. L.L. and V.T. developed the computational pipeline. L.L. created visualizations. E.A.A., N.K.S., L.M.A.P., L.L., and N.J.L.P. contributed to protocol validation and troubleshooting. V.T. and S.J.S. conceived and supervised the work. S.J.S. and V.T. acquired funding.

## Declaration of interests

This work is related to the patent application “Composition comprising MoDIS for DNA modification screening” filed by ETH Zurich, with N.J.L.P., V.T., and S.J.S. as inventors (PCT/EP2024/068922, WO2025012092A1). The patent application covers the design of MoDIS and demonstration of MoDIS-using click-code-seq for sequencing guanine oxidation and AP sites. N.J.L.P. current affiliation is Functional Genomics Center Zurich, Switzerland.

## Declaration of generative AI and AI-assisted technologies in the writing process

During the preparation of this work, the authors used Claude (Anthropic) in order to assist with manuscript drafting, editing, and language refinement, and to assist with the development and debugging of the Click-pipe computational pipeline. After using this tool, the authors reviewed and edited the content as needed and take full responsibility for the content of the publication.
