# Supplementary material for: Click-code-seq protocol for genome-wide single-nucleotide-resolution mapping of major types of DNA modifications
Source: STAR Protoc. 2026 Aug 10;7(3):104776. doi: 10.1016/j.xpro.2026.104776 (PMC13487272; doi:10.1016/j.xpro.2026.104776)
Supplement: Supplementary file 1 [file mmc1.pdf]

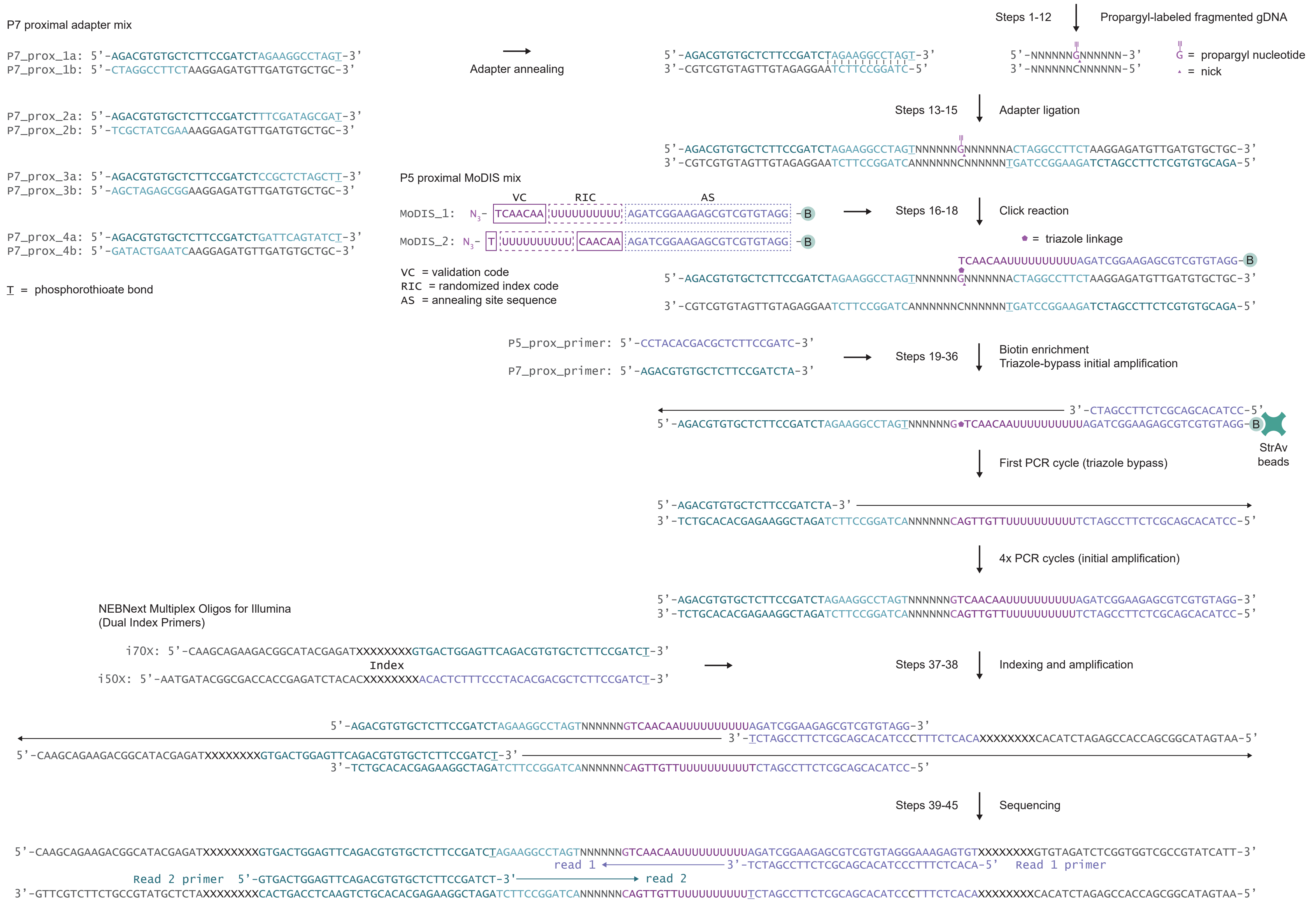

P7 proximal adapter mix
P7_prox_1a: 5'-AGACGTGTGCTCTTCCGATCTAGAAGGCCTAGT-3'
P7_prox_1b: 5'-CTAGGCCTTCTAAGGAGATGTTGATGTGCTGC-3'
P7_prox_2a: 5'-AGACGTGTGCTCTTCCGATCTTTCGATAGCGAT-3'
P7_prox_2b: 5'-TCGCTATCGAAAAGGAGATGTTGATGTGCTGC-3'
P7_prox_3a: 5'-AGACGTGTGCTCTTCCGATCTCCGCTCTAGCTT-3'
P7_prox_3b: 5'-AGCTAGAGCGGAAGGAGATGTTGATGTGCTGC-3'
P7_prox_4a: 5'-AGACGTGTGCTCTTCCGATCTGATTCAGTATCT-3'
P7_prox_4b: 5'-GATACTGAATCAAGGAGATGTTGATGTGCTGC-3'
T = phosphorothioate bond
Adapter annealing
Steps 1-12
Propargyl-labeled fragmented gDNA
5'-AGACGTGTGCTCTTCCGATCTAGAAGGCCTAGT-3'
3'-CGTCGTGTAGTTGTAGAGGAATCTTCCGGATC-5'
5'-NNNNNNGNNNNNN-3'
3'-NNNNNNCNNNNNN-5'
G = propargyl nucleotide
= nick
Steps 13-15
Adapter ligation
5'-AGACGTGTGCTCTTCCGATCTAGAAGGCCTAGTNNNNNNGNNNNNNACTAGGCCTTCTAAGGAGATGTTGATGTGCTGC-3'
3'-CGTCGTGTAGTTGTAGAGGAATCTTCCGGATCANNNNNNCNNNNNNTGATCCGGAAGATCTAGCCTTCTCGTGTGCAGA-5'
P5 proximal MoDIS mix
VC
RIC
AS
MoDIS_1: N3- TCAACAA UUUUUUUUUU AGATCGGAAGAGCGTCGTGTAGG -B
MoDIS_2: N3- T UUUUUUUUUU CAACAA AGATCGGAAGAGCGTCGTGTAGG -B
VC = validation code
RIC = randomized index code
AS = annealing site sequence
Steps 16-18
Click reaction
= triazole linkage
TCAACAAUUUUUUUUUUAGATCGGAAGAGCGTCGTGTAGG-B
5'-AGACGTGTGCTCTTCCGATCTAGAAGGCCTAGTNNNNNNGNNNNNNACTAGGCCTTCTAAGGAGATGTTGATGTGCTGC-3'
3'-CGTCGTGTAGTTGTAGAGGAATCTTCCGGATCANNNNNNCNNNNNNTGATCCGGAAGATCTAGCCTTCTCGTGTGCAGA-5'
P5_prox_primer: 5'-CCTACACGACGCTCTTCCGATC-3'
P7_prox_primer: 5'-AGACGTGTGCTCTTCCGATCTA-3'
Steps 19-36
Biotin enrichment
Triazole-bypass initial amplification
3'-CTAGCCTTCTCGCAGCACATCC-5'
5'-AGACGTGTGCTCTTCCGATCTAGAAGGCCTAGTNNNNNNG•TCAACAAUUUUUUUUUUAGATCGGAAGAGCGTCGTGTAGG-B
StrAv beads
First PCR cycle (triazole bypass)
5'-AGACGTGTGCTCTTCCGATCTA-3'
3'-TCTGCACACGAGAAGGCTAGATCTTCCGGATCANNNNNNCAGTTGTTUUUUUUUUUUTCTAGCCTTCTCGCAGCACATCC-5'
4x PCR cycles (initial amplification)
5'-AGACGTGTGCTCTTCCGATCTAGAAGGCCTAGTNNNNNNGTCAACAAUUUUUUUUUUAGATCGGAAGAGCGTCGTGTAGG-3'
3'-TCTGCACACGAGAAGGCTAGATCTTCCGGATCANNNNNNCAGTTGTTUUUUUUUUUUTCTAGCCTTCTCGCAGCACATCC-5'
NEBNext Multiplex Oligos for Illumina
(Dual Index Primers)
i70X: 5'-CAAGCAGAAGACGGCATACGAGATXXXXXXXXGTGACTGGAGTTCAGACGTGTGCTCTTCCGATCT-3'
Index
i50X: 5'-AATGATACGGCGACCACCGAGATCTACACXXXXXXXXACACTCTTTCCCTACACGACGCTCTTCCGATCT-3'
Steps 37-38
Indexing and amplification
5'-AGACGTGTGCTCTTCCGATCTAGAAGGCCTAGTNNNNNNGTCAACAAUUUUUUUUUUAGATCGGAAGAGCGTCGTGTAGG-3'
3'-TCTAGCCTTCTCGCAGCACATCCCTTTCTCACAXXXXXXXXCACATCTAGAGCCACCAGCGGCATAGTAA-5'
5'-CAAGCAGAAGACGGCATACGAGATXXXXXXXXGTGACTGGAGTTCAGACGTGTGCTCTTCCGATCT-3'
3'-TCTGCACACGAGAAGGCTAGATCTTCCGGATCANNNNNNCAGTTGTTUUUUUUUUUUTCTAGCCTTCTCGCAGCACATCC-5'
Steps 39-45
Sequencing
5'-CAAGCAGAAGACGGCATACGAGATXXXXXXXXGTGACTGGAGTTCAGACGTGTGCTCTTCCGATCTAGAAGGCCTAGTNNNNNNGTCAACAAUUUUUUUUUUAGATCGGAAGAGCGTCGTGTAGGGAAAGAGTGTXXXXXXXXGTGTAGATCTCGGTGGTCGCCGTATCATT-3'
read 1
3'-TCTAGCCTTCTCGCAGCACATCCCTTTCTCACA-5'
Read 1 primer
Read 2 primer
5'-GTGACTGGAGTTCAGACGTGTGCTCTTCCGATCT-3'
read 2
3'-GTTCGTCTTCTGCCGTATGCTCTAXXXXXXXXCACTGACCTCAAGTCTGCACACGAGAAGGCTAGATCTTCCGGATCANNNNNNCAGTTGTTUUUUUUUUUUTCTAGCCTTCTCGCAGCACATCCCTTTCTCACAXXXXXXXXCACATCTAGAGCCACCAGCGGCATAGTAA-5'

Figure S1 - Schematic of the click-code-seq library construction workflow (related to steps 13–37), detailing oligonucleotide sequences and molecular intermediates from adaptor ligation through sequencing.

B, biotin; AS, annealing site sequence; gDNA, genomic DNA; MoDIS, Modified DNA Identifier Sequence; RIC, randomised index code; StrAv, streptavidin; T, phosphorothioate bond; VC, validation code.
